# [^68^Ga]Ga-PSMA-11 PET/CT for monitoring response to treatment in metastatic prostate cancer: is there any added value over standard follow-up?

**DOI:** 10.1186/s13550-019-0554-1

**Published:** 2019-08-29

**Authors:** Jonathan Kuten, David Sarid, Ofer Yossepowitch, Nicola J. Mabjeesh, Einat Even-Sapir

**Affiliations:** 1 Department of Nuclear Medicine, Tel-Aviv Sourasky Medical Center, Sackler School of Medicine, Tel-Aviv University, 6 Weizmann St, 6423906 Tel-Aviv, Israel; 2 Department of Oncology (Uro-Oncology section), Tel-Aviv Sourasky Medical Center, Sackler School of Medicine, Tel-Aviv University, 6 Weizmann St, 6423906 Tel-Aviv, Israel; 3 Department of Urology, Tel-Aviv Sourasky Medical Center, Sackler School of Medicine, Tel-Aviv University, 6 Weizmann St, 6423906 Tel-Aviv, Israel

**Keywords:** [^68^Ga]Ga-PSMA-11, PET/CT, Monitoring, Metastatic, Prostate cancer

## Abstract

**Background:**

The aim of the current study was to assess whether and to what extent monitoring response to treatment using prostate-specific membrane antigen (PSMA)-based positron-emitting tomography/computerized tomography (PET/CT) studies contribute clinically relevant data to routine clinical follow-up during treatment of patients with metastatic prostate cancer (PCa).

**Results:**

Fifty-two patients with metastatic PCa who underwent [^68^Ga]Ga-PSMA-11 PET/CT imaging and serum prostate-specific antigen (PSA) level measurements before and during treatment were investigated. Response was categorized by serum PSA dynamics according to improvement, stable disease, and disease progression and compared to change in imaging findings on pre- and post-treatment PET/CTs. McNemar’s test was used to assess agreement between PET/CT- and PSA-based responses to treatment.

Thirty-four patients (65.4%) had compatible biochemical- and imaging-based response to treatment. However, the imaging and biochemical responses were discrepant in 18/52 patients (34.6%). PET/CT showed progressive disease in 5/52 patients (9.6%) and improvement/stable disease in 13/52 (25%) compared to biochemical assessment results. Discrepancy between imaging and biochemical response was most prominent in biochemically stable patients (90.9%), followed by patients with biochemical progression (33.3%), and in only few (8.7%) patients with biochemical improvement. The imaging-based response was suitable for choosing subsequent treatment in 22 of 30 patients (73.3%) with longer follow-up (median time of 10.3 months (IQR 6.3–18.2)). The relevance of the imaging methodology was reflected by its ability to assess individual lesions in cases of heterogeneous lesion responses, reveal the appearance of new lesions, and identify lesions that required specific consideration, such as targeted radiotherapy.

**Conclusions:**

Results of this retrospective analysis showed that biochemical responses to treatment and [^68^Ga]Ga-PSMA-11 PET/CT-based responses to treatment differ in one third of metastatic PCa patients. The latter additionally enabled lesion-based and not solely patient-based analysis. Monitoring response during treatment by [^68^Ga]Ga-PSMA-11 PET/CT is suitable for decision-making in patient management and choice of treatment in the majority of patients.

## Introduction

Assessment of response to therapy in prostate cancer (PCa) patients relies on a combination of clinical parameters, the biochemical response as reflected by a change in serum prostate-specific antigen (PSA) levels, and on the morphological assessment on computerized tomographic (CT) imaging using the Response Evaluation Criteria in Solid Tumors (RECIST) [1–4]. These measures, however, have limitations. Correlation between PSA levels and disease burden may be hampered in advanced metastatic disease, in patients with visceral metastases not producing PSA, in patients with a heterogeneous response to treatment, and as a result of a flare phenomenon [2]. Using the RECIST criteria for CT may be limited in evaluating small lesions as well as in evaluating response to therapy in skeletal metastases, especially osteoblastic lesions [2, 3]. Few studies have evaluated the use of choline tracers for monitoring response to treatment in PCa patients receiving abiraterone, enzalutamide, and docetaxel, with all showing some discrepancy between the response to treatment as assessed by choline-based positron-emitting tomography (PET/CT) and by biochemical response [5–7].

Small molecule inhibitors of prostate-specific membrane antigen (PSMA) that bind to the extracellular part of the PSMA receptor and then internalize into the PCa cell have been developed and labeled with various radionuclides for PSMA imaging, with [^68^Ga]Ga-PSMA-HBED-CC ([^68^Ga]Ga-PSMA-11) being the most commonly used radiotracer [8–10].

[^68^Ga]Ga-PSMA-11 PET/CT is an effective imaging modality for the staging of intermediate- and high-risk PCa and for the assessment of patients with biochemical failure [8–11]. However, only few initial reports are currently available on the potential role of [^68^Ga]Ga-PSMA-11 PET/CT in monitoring the response to treatment [12–14]. The aim of this study was to retrospectively assess whether and to what extent PSMA PET/CT studies add clinically relevant data to those of routine PSA monitoring during the treatment of patients with metastatic PCa.

## Materials and methods

This retrospective study protocol was approved by the local institutional ethics committee which waived written informed consent (reference ID 0497-18-TLV).

### Patients

Between January 2015 and December 2018, 52 patients had undergone two [^68^Ga]Ga-PSMA-11 PET/CT studies within 6 months and fulfilled the following inclusion criteria: (1) having [^68^Ga]Ga-PSMA-11 avid metastatic disease on the initial PET/CT study and (2) having been treated between the two studies with chemotherapy, radiotherapy, androgen deprivation therapy, radium-223, or a combination of these modalities. The patients’ PSA values were available for correlations. The median interval between the two PET/CT studies was 4.4 months (IQR 3.9–4.9). The patients were initially referred to PET/CT for either staging of intermediate- or high-risk disease (*n* = 9, 17.3%) or because of biochemical failure (*n* = 43, 82.7%) and then referred again for a follow-up scan.

### PET/CT imaging

[^68^Ga]Ga-PSMA-11 was injected intravenously as a bolus at a dose of 148–166.5 MBq between 50–100 min before acquisition was initiated. The patients were instructed to void immediately prior to acquisition. PET/CT studies were performed from the tip of the skull to mid-thigh by means of the Discovery 690 PET/CT system (GE Healthcare). CT acquisition was performed using automatic mA-modulation and 120 kV. CT scans were reconstructed to a slice thickness of 2.5 mm. PET acquisition was performed with acquisition time of 3 min per bed position in 3-D mode. PET images were reconstructed in a matrix size of 128 × 128 with a pixel size of 5.5 mm and slice thickness of 3.3 mm. Reconstruction method was VUE Point FX by GE Healthcare that uses time of flight information and includes a fully 3D OSEM algorithm with 2 iterations, 24 subsets, and filter cutoff of 6.4 mm. VUE Point FX algorithm also includes normalization and image corrections for attenuation, scatter, randoms, and dead time. A standard Z-filter was applied to smooth between transaxial slices.

### Image analysis

All scans were reviewed in consensus by nuclear medicine physicians (EES, JK). Any soft tissue or skeletal lesion showing above normal uptake and not associated with physiological uptake was considered a pathological lesion [15]. Typical pitfalls (i.e., benign and malignant lesions mimicking prostate cancer) in PSMA ligand PET/CT imaging were considered (e.g., ganglia, fractures, sarcoidosis, etc.) [8, 16].

### Response to treatment analysis

A per-patient analysis was performed. Findings on the pre- and post-treatment PET/CT were compared, and “imaging response” was categorized as “progressive disease,” “improved disease (complete or partial response to treatment),” or “stable” disease, according to the following criteria:

- “Progressive disease” was defined as any new lesion, an increase in the size of lesions, or an increase in the intensity and extent of the pathologic uptake.
- “Improvement” was defined as a decrease in the number of lesions, in the intensity and extent of pathologic uptake (partial response), or disappearance of lesions (complete imaging response).
- “Stable disease” was defined as no change in PET/CT findings.
- Mixed-response to treatment was defined as “progressive disease” if any areas of progression were evident and as “improvement” if all areas showed either stable disease or improvement.
- “Imaging response” to treatment was compared to “biochemical response” as determined by serum PSA level (ng/ml) dynamics between pre- and post-treatment values, as previously described [13, 14].
- “Progressive disease” was defined as a rise of ≥ 25% in PSA.
- “Partial response” was defined as a decrease of ≥ 50% in PSA.
- “Stable disease” was defined as any change in PSA between the above thresholds.

### Validation

Clinical follow-up was available for 57% of patients and was used as an outcome standard.

### Statistical analysis

Continuous variables were evaluated for normal distributions using histograms and Q-Q plots. Categorical variables were reported as frequency and percentage, and continuous variables were reported as mean and standard deviation or median and interquartile range (IQR). McNemar’s test was used to assess agreement between PET/CT- and PSA-based response to treatment. Fisher’s exact test, analysis of variance (ANOVA), and Kruskal-Wallis tests were used to compare the characteristics of the patients in whom there was a difference between PET/CT- and biochemical-based response to treatment. All statistical tests were two-sided, and *p* < 0.05 was considered statistically significant. SPSS was used for all statistical analyses (IBM Corp. Released 2017. IBM SPSS Statistics for Windows, Version 25.0. Armonk, NY: IBM Corp)

## Results

Fifty-two patients with metastatic PCa were included in the study. On their initial PET/CT study, 29 patients (55.8%) had pathologic uptake in their prostate or prostate bed, 32 (61.5%) had bone metastases, 42 (80.8%) had nodal disease, two had liver metastases (3.8%), two had peritoneal implants (3.8%), and one (1.9%) had lung metastases. Twenty-four patients (46%) had metastatic disease to more than one organ (most commonly skeletal and lymph-nodes). Twenty-three patients (44%) received combination therapy (hormonal treatment and salvage radiotherapy being the most common). Table 1 summarizes the pathological findings observed on the initial PET/CT study and the treatment given between the two PET/CT studies.

**Table 1:** Patient characteristics

Parameter	Value
Total number of patients	52
Age, years (mean ± SD)	71.5 ± 9
PSA at first PET/CT (ng/ml), median (IQR)	6.6 (1.3–28.3)
PSA at second PET/CT (ng/ml), median (IQR)	1.6 (0.3–12.1)
Gleason score (*n*)	
6	6 (11.5%)
7	2 (3.8%)
7 (3 + 4)	8 (15.4%)
7 (4 + 3)	6 (11.5%)
8	9 (17.3%)
9	12 (23.1%)
10	2 (3.8%)
n/a	7 (13.5%)
Disease extent demonstrated on first PET/CT (*n*)	
Prostatic disease	29 (55.8%)
Bone metastases	32 (61.5%)
Lymph nodes	42 (80.8%)
Liver metastases	2 (3.8%)
Peritoneal implants	2 (3.8%)
Lung metastases	1 (1.9%)
Treatment received between the two PET/CT scans (*n*)	
Hormonal therapy	42 (80.8%)
Salvage radiotherapy	17 (32.7%)
Chemotherapy	12 (23.1%)
Radium-223	2 (3.8%)

### PET/CT- vs. PSA-based depiction of response to treatment

**Fig. 1:**
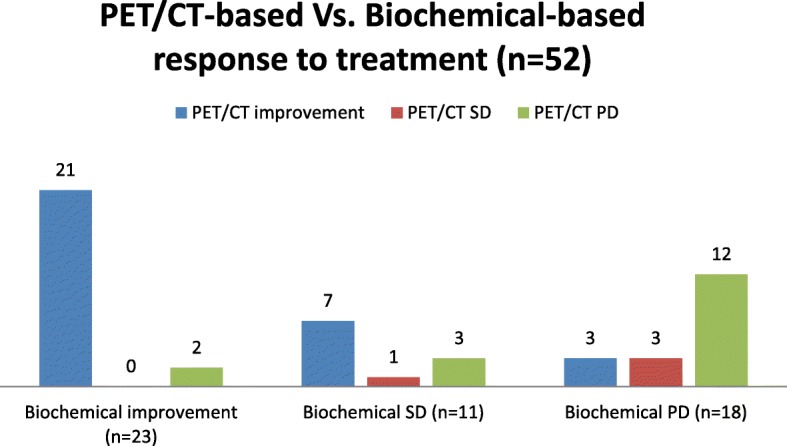
PET/CT-based vs. biochemical-based response to treatment (*n* = 52)

Thirty-four of the 52 study patients (65.4%) had compatible biochemical- and imaging-based response to treatment. Twenty-one improved (40.4%), 12 progressed (23.1%), and one was stable (1.9%) according to PET/CT findings. Twenty-three patients (44.2%) had partial response to treatment, 11 (21.2%) had stable disease, and 18 (34.6%) had progressive disease according to biochemical evidence. Thirty-one patients (59.6%) had improvement or partial or complete response, 4 (7.7%) had stable disease, and 17 (32.7%) had progressive disease according to PET/CT findings. There were discrepancies in responses assessed by PET/CT and those assessed by biochemical response for 18 patients (34.6%): 5 (9.6%) had progressed and 13 (25%) improved or had stable disease on PET/CT findings compared to the biochemical response (Fig. 1).

Discrepancy was most prominent for biochemically stable patients, with 10 of 11 (90.9%) PET/CT scans showing progression (3 patients) or improvement (7 patients).

Interestingly, the disease in one third of patients with biochemical progression was stable or even improved on PET/CT. The smallest degree of discrepancy was found among the 23 patients with biochemical improvement, where PET/CT also showed improvement in 21 of them (91.3%). The difference between PET/CT-based and biochemical-based assessment of response to treatment had a tendency towards significance (*p* = 0.066), but it did not reach a level of significance, probably owing to the small number of patients included in the study. This difference was not associated with any of the patient characteristics, including age, Gleason score, PSA values at the time of initial or follow-up PET/CT scans, any specific treatment received, or having metastatic disease.

The difference in the assessment of response to treatment was more prominent for patients referred for PET/CT due to biochemical failure, for whom post-treatment PET/CT and PSA dynamics showed a different response in 16/43 patients (37.2%), but only in 2/9 (22.2%) patients who had been initially referred for staging

### Impact of follow-up PET/CT on patient management

**Fig. 2:**
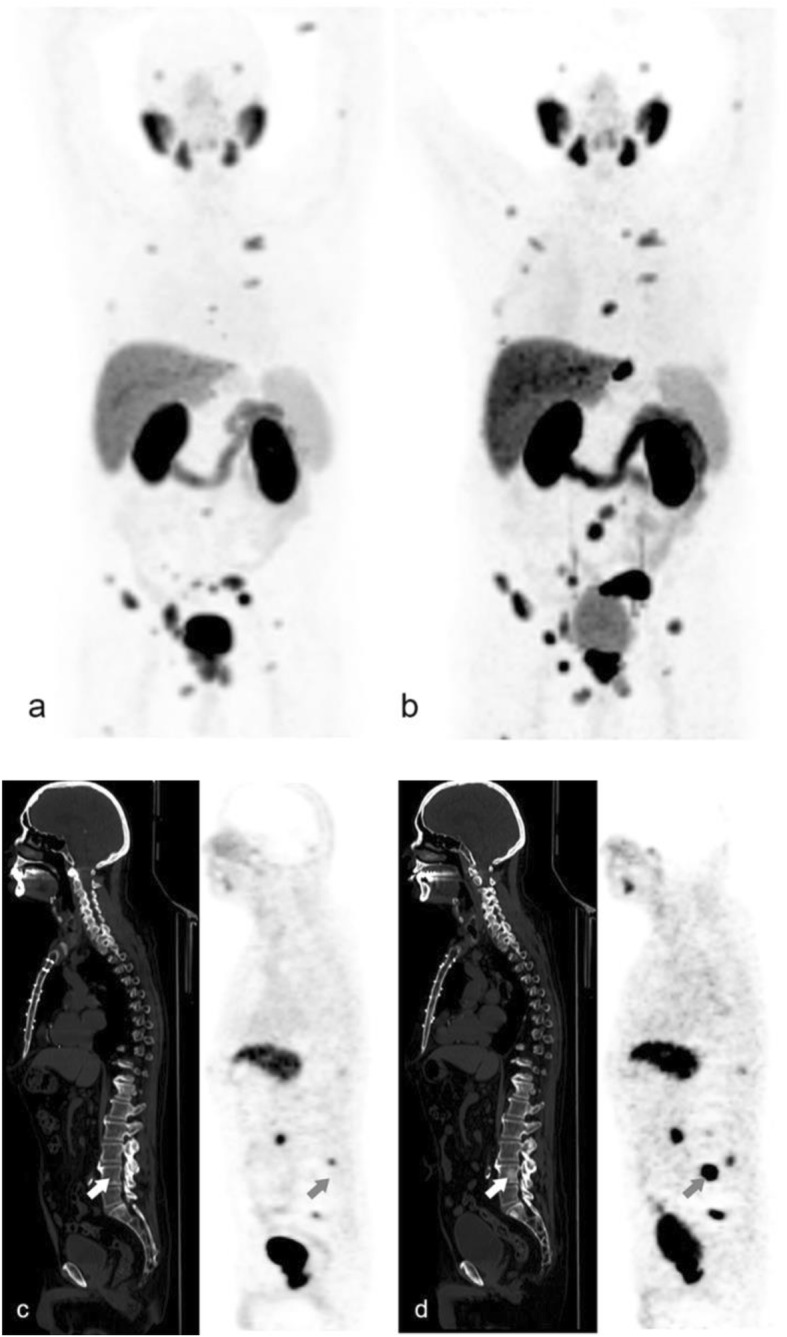
A 77-year-old patient with PCa, Gleason score 9, and PSA 13.5 ng/ml. **a** [^68^Ga]Ga-PSMA-11 PET/CT performed for staging showed extensive local disease as well as metastatic disease to pelvic lymph nodes and numerous skeletal metastases. **b** PSA levels declined by 25% to 10.8 ng/ml after 4.5 months of treatment with abiraterone (biochemical stable disease). Follow-up PET/CT showed stable disease in soft tissue, however, marked progression was noted in bone, with more extensive known lesions as well as new lesions. Imaging response was categorized as “progressive-disease.” The arrows in **c** and **d** indicate an example of a new PSMA-avid sclerotic bone lesion. This patient was continued on the same treatment and had marked progression on a recent follow-up PET as well as elevation of serum PSA to 36 ng/ml

Clinical follow-up was available for 30 of the 52 study patients (57.7%), with a median time of 10.3 months (IQR 6.3–18.2). There appeared to be discrepant responses between PET/CT and biochemical assessment in 11 patients (Table 2). The response as demonstrated on PET/CT guided the clinical management of 9 of those 11 patients (82%). In six patients, the PET/CT-based response suggested improvement or no evidence of disease, whereupon surveillance or continuation of hormonal therapy was the preferred treatment approach, while the PET/CT findings in three of four patients with stable or improved PSA levels revealed disease progression, resulting in a more aggressive treatment approach and/or targeted radiotherapy of lesions requiring special attention. Although there was agreement between PET/CT and biochemical assessment in an additional 11 of the 30 patients, the PET/CT data were taken as being more contributory for treatment tailoring in patients with mixed response (Fig. 2) by highlighting disease sites that did not respond to treatment and needed a targeted treatment approach.

**Table 2:** Management of patients with a discrepant response on PET/CT and their biochemical assessment

Patient	Indication for first PET/CT	Findings on first PET/CT	Treatment between PET/CTs	Findings on second PET/CT	PET/CT response	Biochemical response	Management and outcome
1	Staging	Prostatic lesion, metastatic disease to bone and liver	Hormonal	Improvement in bone and liver disease, stable disease in the prostate	↓	↑	Continued same hormonal therapy
2	BF	Prostatic lesion, pelvic nodal involvement	Hormonal + radiation therapy to prostate and lymph nodes	NED	↓	↔	Continued same hormonal therapy, with further biochemical improvement
3	BF	Prostatic lesion seminal vesicles, pelvic lymph nodes, and bone	Hormonal	Stable disease in the prostate, seminal vesicles and lymph nodes, more extensive bone disease	↑	↔	Referred to LU177-PSMA
4	BF	Pelvic nodal disease	SBRT to lymph nodes	NED	↓	↔	Surveillance only
5	BF	Prostatic lesion, lymph nodes, lung and bone	Hormonal + chemotherapy	More extensive uptake in the prostate, enlargement of involved lymph nodes, lung mets, heterogeneous response in bone with new bony mets	↑	↔	Palliative radiation therapy and abiraterone
6	BF	Prostatic and rib lesions	Hormonal + SBRT to a rib	Stable disease in the prostate, no uptake in rib	↓	↔	Radiation to the prostate, hormonal therapy with temporary remission
7	BF	Prostatic lesion, involved pelvic lymph nodes and bone metastases	Hormonal + SBRT to L2 + XOFIGO	Stable disease in the prostate and lymph nodes, progression in known bone mets. L2 needed special attention	↑	↓	SBRT to L2 vertebra
8	BF	Involved pelvic lymph node	SBRT to LN	NED	↓	↔	Surveillance only
9	BF	Involved lymph nodes and bone mets	Hormonal	Reduction in size of nodes and decreased in uptake of bone mets	↓	↔	Continued same hormonal therapy with further biochemical improvement
10	BF	Involved retroperitoneal lymph node	SBRT to lymph node	Finding unchanged	↔	↑	Surveillance only on follow up PET/CT disease progression
11	BF	Prostatic lesion, seminal vesicles, pelvic lymph nodes and bone mets	Hormonal	Stable disease in the prostate and seminal vesicles, Increase in size of one involved lymph node and more extensive bone disease	↑	↔	Continued same hormonal therapy. Progression on follow-up PET/CT

Response to treatment: ↓ improvement; ↑ progressive disease (PD); ↔ stable disease (SD)

*Abbreviations*: *SBRT* stereotactic body radiation therapy, *BF* biochemical failure, *NED* no evidence of disease, *mets* metastases

## Discussion

The role of [^68^Ga]Ga-PSMA-11 PET/CT in monitoring response to treatment in PCa remains unclear due to the lack of relevant data, with few reports having addressed this issue. Baumann et al. showed a high rate of metabolic response of PSMA PET-positive metastatic lesions after hypofractionated radiotherapy [12]. Seitz et al. reported a higher level of agreement, albeit non-significant, between PET and biochemical response to treatment than that between RECIST 1.1 CT and biochemical response in 23 patients treated with docetaxel [13]. Schmidkonz et al. [14] and Schmuck et al. [17] evaluated [^68^Ga]Ga-PSMA-11 PET/CT whole-body-derived metabolic parameters for monitoring response to treatment in PCa. Both of those studies demonstrated a correlation between metabolic parameters and biochemical response, as well as a potential superiority of these parameters over CT in assessing response to treatment.

The findings of the current study revealed that biochemical-based and [^68^Ga]Ga-PSMA-11 PET/CT-based assessment of response to treatment may differ in one third of metastatic PCa patients. Monitoring response to therapy by PET/CT resulted in progressive disease in 9.6% and improvement/stable disease in 25% of the patients. The most prominent discrepancy between PET/CT imaging and measurement of PSA levels was in patients with biochemical determination of stable disease: PET/CT findings suggested either improvement or progressive disease in 90.9% of them. This discrepancy between imaging and biochemical follow-up is built-in, given that PSA levels provide a global impression of disease extent while imaging involves lesion-based assessment.

Our analysis revealed that patients with biochemically determined stable disease or improved PSA levels may have heterogeneous responses, which may indicate improvement of some lesions while other lesions may progress and new lesions may sometimes be present and indicate progressive disease. In contrast, [^68^Ga]Ga-PSMA-11 PET/CT suggested improvement earlier than evidence of improvement in PSA levels which may appear later without additional therapy in some patients with biochemically stable disease. This was the case in patients who were evaluated after stereotactic body radiation therapy.

Clinical follow-up was available for 30 patients as outcome standard. In 22 of them (73.3%), imaging data guided further therapeutic management. Notably, PET/CT and biochemical assessment suggested different responses in 11 of them.

A major added value of imaging in monitoring response is that it allows lesion-based and not only patient-based analysis. Progression of some lesions may be obscured by a favorable response of other lesions, resulting in stable PSA levels in patients with heterogeneous lesion responses. Imaging of lesions may better assist in risk stratification and identification of lesions requiring special attention, as well as for tailoring of the radiation field.

As the costs associated with PSMA imaging are substantially higher than PSA measurement, the former should be judiciously used when clinically-relevant, e.g., when post-treatment clinical assessment and biochemical response are discrepant, as well as whenever targeted therapy to some diseased areas is considered.

The major limitation of this study is its retrospective nature. In addition, the study population was both small and heterogeneous in terms of the provision of treatments. Clinical follow-up served as outcome standard and was not available for all patients due to the retrospective nature of this work. The PET-based response to treatment was compared to the biochemical response alone, and not to other imaging modalities. Information on the latter was unavailable, since it has become common practice for clinicians to rely on PSMA PET/CT as a stand-alone imaging method. Indeed, it is the most sensitive modality currently available for evaluating the extent of disease in PCa.

## Conclusions

The results of this retrospective analysis demonstrated that the biochemical response to treatment assessment and the [^68^Ga]Ga-PSMA-11 PET/CT–based response assessment differed in one third of patients with metastatic PCa. This discrepancy was often the result of the ability of imaging to allow for lesion-based and not solely patient-based analysis. Monitoring response to treatment by [^68^Ga]Ga-PSMA-11 PET/CT was more suitable for treatment management in a large proportion of patients.

## Data Availability

The datasets used and/or analyzed during the current study are available from the corresponding author on reasonable request.

## Abbreviations

BF: Biochemical failure
CT: Computed tomography
IQR: Interquartile range
Mets: Metastases
NED: No evidence of disease
PCa: Prostate cancer
PD: Progressive disease
PET/CT: Positron-emitting tomography/computerized tomography
PSA: Prostate-specific antigen
PSMA: Prostate-specific membrane antigen
RECIST: Response Evaluation Criteria in Solid Tumors
SBRT: Stereotactic body radiation therapy
SD: Stable disease

## References

[1] Mottet N, Bellmunt J, Bolla M, Briers E, Cumberbatch MG, De Santis M (2017). EAU-ESTRO-SIOG Guidelines on Prostate Cancer. Part 1: Screening, Diagnosis, and Local Treatment with Curative Intent. Eur Urol..

[2] Ceci F, Herrmann K, Hadaschik B, Castellucci P, Fanti S (2017). Therapy assessment in prostate cancer using choline and PSMA PET/CT. Eur J Nucl Med Mol Imaging..

[3] Fech V, Alberts I, Rominger A, Afshar-Oromieh A. PSMA-ligand PET allows a more accurate therapeutic response evaluation of bone metastases in prostate cancer compared to computed tomography. Nuklearmedizin. 2019. 10.1055/a-0895-5078. 10.1055/a-0895-5078 31083752

[4] Eisenhauer EA, Therasse P, Bogaerts J, Schwartz LH, Sargent D, Ford R (2009). New response evaluation criteria in solid tumours: revised RECIST guideline (version 1.1). Eur J Cancer..

[5] Maines F, Caffo O, Donner D, Sperduti I, Bria E, Veccia A (2016). Serial 18F-choline-PET imaging in patients receiving enzalutamide for metastatic castration-resistant prostate cancer: response assessment and imaging biomarkers. Futur Oncol..

[6] Ceci F, Castellucci P, Graziani T, Schiavina R, Renzi R, Borghesi M (2016). 11C-Choline PET/CT in castration-resistant prostate cancer patients treated with docetaxel. Eur J Nucl Med Mol Imaging..

[7] De Giorgi U, Caroli P, Burgio SL, Menna C, Conteduca V, Bianchi E, et al. Early outcome prediction on 18F-fluorocholine PET/CT in metastatic castration-resistant prostate cancer patients treated with abiraterone. Oncotarget. 2015;5(23). 10.18632/oncotarget.2558. 10.18632/oncotarget.2558 PMC4322993 25504434

[8] Afshar-Oromieh A, Holland-Letz T, Giesel FL, Kratochwil C, Mier W, Haufe S (2017). Diagnostic performance of 68Ga-PSMA-11 (HBED-CC) PET/CT in patients with recurrent prostate cancer: evaluation in 1007 patients. Eur J Nucl Med Mol Imaging.

[9] Hofman MS, Hicks RJ, Maurer T, Eiber M (2018). Prostate-specific membrane antigen PET: clinical utility in prostate cancer, normal patterns, pearls, and pitfalls. RadioGraphics..

[10] Caroli P, Sandler I, Matteucci F, De Giorgi U, Uccelli L, Celli M (2018). 68Ga-PSMA PET/CT in patients with recurrent prostate cancer after radical treatment: prospective results in 314 patients. Eur J Nucl Med Mol Imaging.

[11] Zhang J, Shao S, Wu P, Liu D, Yang B, Han D (2019). Diagnostic performance of 68Ga-PSMA PET/CT in the detection of prostate cancer prior to initial biopsy: comparison with cancer-predicting nomograms. Eur J Nucl Med Mol Imaging.

[12] Baumann René, Koncz Mark, Luetzen Ulf, Krause Fabian, Dunst Juergen (2017). Oligometastases in prostate cancer. Strahlentherapie und Onkologie.

[13] Seitz Anna Katharina, Rauscher Isabel, Haller Bernhard, Krönke Markus, Luther Sophia, Heck Matthias M., Horn Thomas, Gschwend Jürgen E., Schwaiger Markus, Eiber Matthias, Maurer Tobias (2017). Preliminary results on response assessment using 68Ga-HBED-CC-PSMA PET/CT in patients with metastatic prostate cancer undergoing docetaxel chemotherapy. European Journal of Nuclear Medicine and Molecular Imaging.

[14] Schmidkonz Christian, Cordes Michael, Schmidt Daniela, Bäuerle Tobias, Goetz Theresa Ida, Beck Michael, Prante Olaf, Cavallaro Alexander, Uder Michael, Wullich Bernd, Goebell Peter, Kuwert Torsten, Ritt Philipp (2018). 68Ga-PSMA-11 PET/CT-derived metabolic parameters for determination of whole-body tumor burden and treatment response in prostate cancer. European Journal of Nuclear Medicine and Molecular Imaging.

[15] Rauscher I, Maurer T, Fendler WP, Sommer WH, Schwaiger M, Eiber M (2016). 68Ga-PSMA ligand PET/CT in patients with prostate cancer: How we review and report. Cancer Imaging..

[16] Sheikhbahaei S, Afshar-Oromieh A, Eiber M, Solnes LB, Javadi MS, Ross AE (2017). Pearls and pitfalls in clinical interpretation of prostate-specific membrane antigen (PSMA)-targeted PET imaging. Eur J Nucl Med Mol Imaging.

[17] Schmuck Sebastian, von Klot Christoph A., Henkenberens Christoph, Sohns Jan M., Christiansen Hans, Wester Hans-Jürgen, Ross Tobias L., Bengel Frank M., Derlin Thorsten (2017). Initial Experience with Volumetric68Ga-PSMA I&T PET/CT for Assessment of Whole-Body Tumor Burden as a Quantitative Imaging Biomarker in Patients with Prostate Cancer. Journal of Nuclear Medicine.

